# Development of Bio-Inspired Hierarchical Fibres to Tailor the Fibre/Matrix Interphase in (Bio)composites

**DOI:** 10.3390/polym13050804

**Published:** 2021-03-05

**Authors:** Estelle Doineau, Bernard Cathala, Jean-Charles Benezet, Julien Bras, Nicolas Le Moigne

**Affiliations:** 1 Polymers Composites and Hybrids (PCH), IMT Mines Alès, 30100 Alès, France; doineau.estelle@orange.fr (E.D.); jean-charles.benezet@mines-ales.fr (J.-C.B.); 2 Institute of Engineering, Université Grenoble Alpes, CNRS, Grenoble INP, LGP2, 38000 Grenoble, France; julien.bras@grenoble-inp.fr; 3 INRAE, UR BIA, 44316 Nantes, France

**Keywords:** hierarchical fibre, nano-objects deposition, interphase, composite

## Abstract

Several naturally occurring biological systems, such as bones, nacre or wood, display hierarchical architectures with a central role of the nanostructuration that allows reaching amazing properties such as high strength and toughness. Developing such architectures in man-made materials is highly challenging, and recent research relies on this concept of hierarchical structures to design high-performance composite materials. This review deals more specifically with the development of hierarchical fibres by the deposition of nano-objects at their surface to tailor the fibre/matrix interphase in (bio)composites. Fully synthetic hierarchical fibre reinforced composites are described, and the potential of hierarchical fibres is discussed for the development of sustainable biocomposite materials with enhanced structural performance. Based on various surface, microstructural and mechanical characterizations, this review highlights that nano-objects coated on natural fibres (carbon nanotubes, ZnO nanowires, nanocelluloses) can improve the load transfer and interfacial adhesion between the matrix and the fibres, and the resulting mechanical performances of biocomposites. Indeed, the surface topography of the fibres is modified with higher roughness and specific surface area, implying increased mechanical interlocking with the matrix. As a result, the interfacial shear strength (IFSS) between fibres and polymer matrices is enhanced, and failure mechanisms can be modified with a crack propagation occurring through a zig-zag path along interphases.

## 1. Introduction

By combining biopolymers and minerals into hierarchical nanoscaled structures, nature succeeds in developing hybrid materials with amazing mechanical performances such as high strength toughness adapted to the specific needs of biological systems [1]. In this respect, complex biological architectures, displaying self-assembly processes and implying the key role of nanostructuration and nano-objects intrigue researchers and inspire them for the development of innovative engineering materials [2,3,4,5,6,7]. Elaboration of bio-inspired materials has already been investigated in a plethora of engineering materials, mimicking natural systems such as nacre, tooth, bone, or wood [8,9,10,11,12]. Practically, it seems that these architectures modify stress transfer mechanisms within the material and boost their strength and fracture toughness thanks to nanostructuration and the development of a “hierarchical architecture” [13,14,15,16,17]. The hierarchical architecture of a system can be defined as the deployment of structures exhibiting specific organizations at different length scales, going from the macro- to the nanoscale, and ensuring interesting properties to the entire material. This concept has notably been used in composite materials with the implementation of hierarchical fibres via the deposition of nano-objects on fibre surfaces. As an example, the whiskerization of carbon fibres with carbon nanotubes (CNTs) has been developed for the manufacturing of carbon fibre reinforced composites [18,19]. The developed nanostructured composites displayed enhanced mechanical properties due to increased mechanical interlocking and lower local stress concentrations at the fibre/matrix interface, hence resulting in higher strength and toughness [20,21,22,23,24]. Karger-Kocsis et al. (2015) also pointed out the potential of such hierarchical composites for sensing applications, that is, the in-situ sensing of stress, strain, and damage for structural health monitoring [18].

Besides, current environmental issues push towards the implementation of eco-friendly and high-performance composite materials on the market, either hybrid, that is, synthetic/bio-based, or fully bio-based and reinforced with natural fibres and/or bio-based nano-objects [25,26]. In this regard, the development of hierarchical fibres at the interphase zone in (bio)composites is at its very early stage and could be an interesting strategy to tackle current and future challenges raised by the implementation of fully bio-based natural fibre reinforced biocomposites in industrial applications [27,28,29].

This review reports on the current state of the art use of hierarchical fibres for improving fibre/matrix interfacial adhesion and toughening of (bio)composite materials. First, several biological systems will be described to understand the role of nano-objects in naturally occurring hierarchical architectures. Then, current studies on hierarchical fibre reinforced composites will be discussed and divided into three main categories: (i) fully synthetic hierarchical composites, (ii) hybrid hierarchical composites either reinforced with bio-based nanoparticle modified synthetic fibres, or with synthetic or mineral nanoparticle modified natural fibres, and (iii) hierarchical biocomposites reinforced with bio-based nanoparticle modified natural fibres, the matrix being oil-based or bio-based.

## 2. Naturally Occurring Hierarchical Structures: Towards the Conception of Bio-Inspired Architectures for Composite Materials

### 2.1. Hierarchical Structures in Biological Systems

The complex architectures found in naturally occurring biological systems are the result of billions of years of evolution with continuous refining of their structure to face different challenges and adapt in an ever-changing environment. They are made of hierarchical micro/nanostructures with soft and organic interfaces (or matrices) and small stiff building blocks. In general, these hierarchical structures include nano-objects, enhancing drastically the mechanical properties, as for instance in bones [30,31], nacre of seashells [32,33], or wood [34].

**Figure 1:**
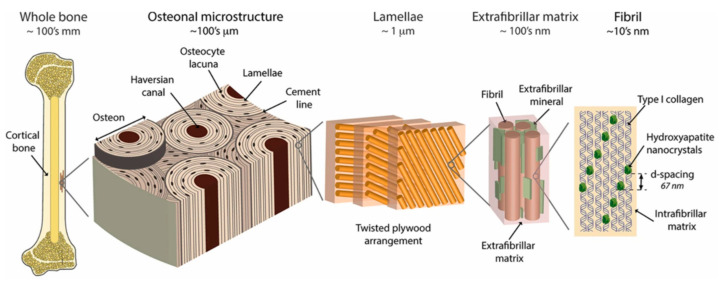
Hierarchical structure of human cortical bone with the presence of hydroxyapatite mineral nanocrystals (reprinted with permission from Zimmermann et al., 2016 [40]).

The bone is structured by mineral crystals, that is, hydroxyapatite nanocrystals (thickness 2–4 nm; length up to 100 nm), embedded in a (collagen-rich) protein matrix [35,36], as illustrated in Figure 1. The specific three-dimensional network of hydroxyapatite nanocrystals embedded into collagen fibrils shows peculiar deformation mechanisms that impact positively the mechanical properties of bones [13,14]. Indeed, collagen fibrils are assembled into collagen fibres, hence forming macroscopic structures such as osteons and lamellae. This hierarchical structure developed over the entire system induces crack deflection and crack bridging mechanisms with impressive properties such as self-healing and adaptation to local stress [37,38,39].

The nanostructuration plays a central role in the mechanical behaviour of bones. Gao et al. (2003) reported that mineral nanocrystals in natural materials have an optimum size to ensure optimum fracture strength and maximum tolerance of flaws for toughness [41]. Indeed, according to the Griffith criterion, when the mineral size exceeds a critical length of about 30 nm, the fracture strength is sensitive to crack-like flaws and fails by stress concentration. As the mineral platelets size drops below this critical length, the mineral behaves similarly to perfect crystals whatever the type of pre-existing flaws due to accidental soft protein matrix incorporation or defective crystal structure. Considering the bone material, nanometric dimensions of hydroxyapatite crystals and their hierarchical structure thus appears as an optimization for better reinforcement.

**Figure 2:**
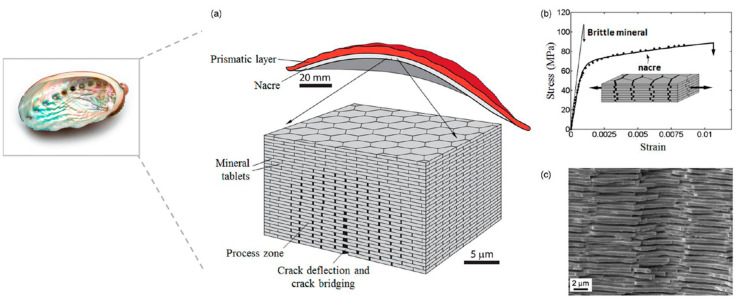
Nacre from mollusc shell (**a**) three-dimensional nanostructure of nacre organized in brick wall fashion, (**b**) tensile stress-strain curves for pure aragonite and nacre, (**c**) SEM image of the fracture surface of nacre with crack propagation deflected along the interfaces (reprinted with permission from Barthelat et al., 2015 [4]).

Nacre can be found in the inner layer of mollusc shells (mussels, oysters, etc.) and is made of aragonite plate-like crystals (thickness 200–500 nm; length few micrometres) and a soft matrix of proteins and polysaccharides present in very small amounts [42]. These mineral platelets are structured in a three-dimensional brick wall fashion, as illustrated in Figure 2a. The interfaces between each platelet are soft and very thin (30–40 nm) but seem to play a key role in the toughening mechanisms of mollusc shells. In fact, nacre has a fracture toughness around 3000 times higher than its main component, that is, the aragonite CaCO_3_ [33], due to its high deformation capacity when submitted to stress along the direction of the platelets (Figure 2b). A sliding of platelets relative to each other occurs under stress, this phenomenon is driven by the nanostructured interfaces, strengthened by the nano-asperities of the platelets surface, and controlled by the hydration rate [43,44]. Moreover, the crack propagation in nacre is deflected along the interfaces in Figure 2c, which strongly increases the fracture toughness of the material [1].

**Figure 3:**
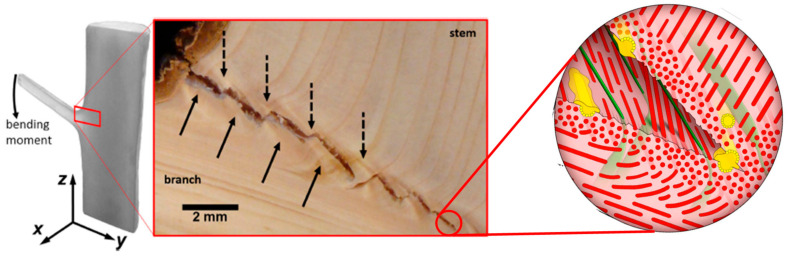
Zig-zag cracking of branch-stem interface with the model (insert) of the crack propagation in the sacrificial tissue. Wood rays (green) reinforced with tracheids (red) form tissue bundles responsible for crack bridging. High concentration of resin ducts (yellow) activated after the cracking ensures antimicrobial and hydrophobic protection. (reprinted with permission from Müller et al., 2015 [15]).

Plant cell walls are the main components of annual plant stems and wood ensuring structural, conducting functions, and protection against pathogens. They are complex composite materials with a hierarchical structure starting from the stem or branch down to the cellulose elementary fibrils/crystallites (thickness 3–5 nm; length 100–1000 nm) associated with various biopolymers as hemicelluloses, lignin and pectins in various amount depending on the species [45]. Müller et al. studied the macroscopic biological interface branch-stem of a Norway spruce in terms of microstructure, and mechanical and self-healing mechanisms [15]. As illustrated in Figure 3, they observed during the bending and breakage of the branch, the occurrence of a zig-zag crack propagation path with crack bridging at the branch-stem interface. This zig-zag shape of cracks has also been observed in natural materials like nacre and bone and requires much more energy for the formation and extension of cracks [46]. This crack pattern is the consequence of the complex and optimized hierarchical interface branch-stem with multiple length scales. At the nanometric scale, the cell orientation and the microfibrillar angle (MFA) of cellulose microfibrils within plant cell walls are perfectly adjusted in tissues of the branch and stem to ensure high flexibility and strength. Moreover, the distribution of cells appears to be adapted to the local damages that could occur at the branch-stem interface, by limiting local stress concentrations.

The description of naturally occurring hierarchical architectures and their behaviour under stress can thus be a model for the structuration of interfaces in man-made composite materials based on hierarchical structure concepts using nano-objects. The next section will focus on the conception of hierarchical fibre reinforced composite materials implying the use of nano-objects, as observed in the biological systems presented previously. The creation of such hierarchical composites targets the enhancement of the fibre/matrix interfacial adhesion, a key parameter for stress transfer in composite materials.

### 2.2. Towards the Conception of Hierarchical Composite Materials Using Nano-Objects

Fibre-reinforced polymer composites are commonly used in many daily life applications and consist of at least three phases: the polymer matrix (thermoplastic, thermoset), the reinforcing fibres (glass, carbon, natural fibres, etc.) and interfacial zones in between, also called interphases since they develop over a certain thickness from the bulk of the matrix to the fibres. The matrix protects reinforcing fibres and achieves the distribution of loads to the fibres and among fibres through the interphases when the composite is submitted to mechanical solicitations [18,47]. The mechanical properties of the composite such as strength and stiffness are primarily determined by the reinforcement characteristics (intrinsic mechanical properties, volume fraction, orientation, L/d aspect ratio, i.e., L is the fibre length and d corresponds to the fibre lateral dimensions), but the characteristics of the interphases also appear as a key. Indeed, this three-dimensional region between the fibres and the matrix can ensure the load transfer from the matrix to the fibres, provided that the fibre/matrix interfacial adhesion is good enough. Thereby, the bonding strength at the interface largely influences the final properties of the composite, and the role of the interfacial adhesion on their structural integrity is now commonly accepted.

One of the main challenges when developing composite materials is precisely to combine strength and toughness [48]. In general, a strong fibre/matrix interfacial adhesion will achieve high strength and stiffness, while a weaker interfacial adhesion or flexible interphase could enhance toughness performance. Interestingly, structural natural materials such as bones, shells and plant stems seem to succeed in gathering these mechanical properties often antagonistic, and it appears that the combination of soft and stiff components inside the systems, in optimized architectures and concentrations, could be the key for such reinforcement [4,49]. Moreover, considering the three examples of biological systems described above, the presence of nano-objects within the structure is likely to reinforce the most vulnerable parts of the system undergoing higher stress levels [41]. By now, if we transpose all these observations of natural materials to synthetic and man-made composite materials, the concept of hierarchical architecture appears as an attractive strategy to tailor the fibre/matrix interphase zone, and so increase the strength and toughness and hamper crack propagation within composites.

**Figure 4:**
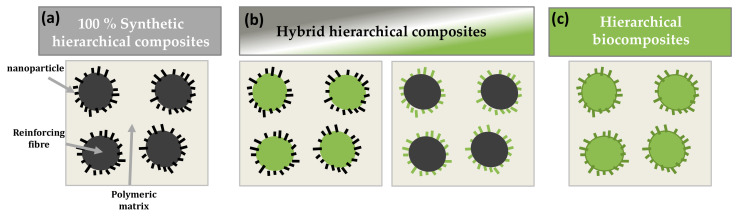
Schematic structures of the different hierarchically nanostructured fibre reinforced composites with: (**a**) fully synthetic hierarchical composites, (**b**) hybrid hierarchical composites either reinforced with bio-based nanoparticle modified synthetic fibres or with synthetic or mineral nanoparticle modified natural fibres; and (**c**) hierarchical biocomposites reinforced with bio-based nanoparticle modified natural fibres. Green colour stands for bio-based (nano-)reinforcements and black colour stands for synthetic (nano-)reinforcements.

The following sections will focus on the different types of hierarchically nanostructured fibre reinforced composites as schematized in Figure 4, the matrix being either oil-based or bio-based.

## 3. Hierarchical Interphase in Fully Synthetic and Hybrid Fibre Reinforced Composites

### 3.1. Fully Synthetic Hierarchical Fibre Reinforced Composites

**Figure 5:**
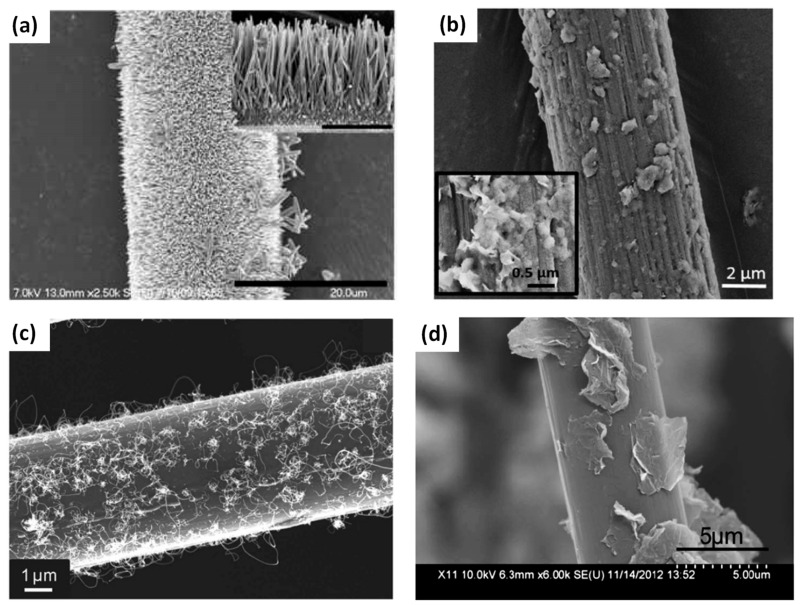
SEM images of hierarchical synthetic fibres (**a**) aligned ZnO nanowires coated and grown on aramid fibres (functionalized with carboxylic acid groups); (**b**) Si-nanoclays (nanoclays functionalized with APTES) grafted on N-CF (carbon fibre modified with GTMAC); (**c**) CNTs grown on oxidized IM7 carbon fibres using CVD method; (**d**) graphene nanosheets deposited on carbon fibre at a content of 1 wt% (reprinted with permission from Ehlert and Sodano 2009 [56], Zabihi et al., 2017 [50], Qian et al., 2010 [57] and Chen et al., 2015 [21]).

The concept of developing hierarchically nanostructured fibres has been well studied in synthetic fibre reinforced composites, especially in carbon and glass fibre composites with the deposition of nanofillers such as nanoclays (layered silicate), carbon nanotubes (CNTs, single, double, and multi-walled), graphene and zinc oxide (ZnO) nanowires [16,18,19,20,50,51,52]. The anchoring of carbon nanofillers on the fibre surface can be achieved by two main pathways [18,19]: (i) chemical reaction between nanofillers and fibres (sizing, electrospraying, electrostatic, and electrophoretic deposition, chemical grafting, etc.) [53,54] or (ii) direct growth of nanofillers on fibre surface also called “whiskerization” with various Chemical Vapour Deposition (CVD) methods (thermal, injection, etc.) [55]. Besides, ZnO nanowires were grown on the surface of aramid and carbon fibres thanks to the strong interactions between ZnO and carboxylic acid functional groups added on fibre surfaces [16,17,56]. Some examples of such synthetic hierarchical fibres are presented in Figure 5.

**Figure 6:**
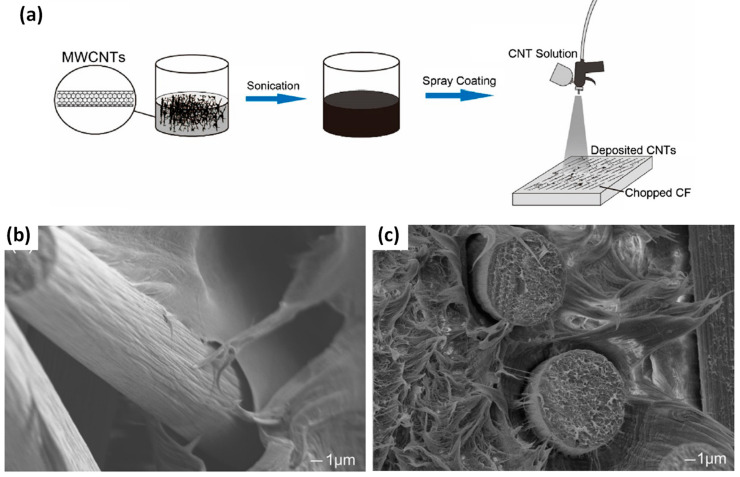
(**a**) schematic for the spraying coating of CNT onto carbon fibres, Fracture surfaces for (**b**) pristine carbon fibre/HDPE composites and (**c**) CNTs coated carbon fibre/HDPE composites (reprinted with permission from Hu et al., 2019 [58]).

First, the creation of hierarchical fibres by the growth or deposition/grafting of nanofillers at their surface induces modifications in the fibre surface topography. Zabihi et al. (2017) studied the grafting of (3-aminopropyl) triethoxysilane (APTES) functionalized nanoclays on glycidyltrimethylammonium chloride (GTMAC) modified carbon fibres [50]. They obtained hierarchical carbon fibres, as shown in Figure 5b, with increased surface root mean square roughness from ~64 nm to ~103 nm as measured by atomic force microscopy (AFM) on 1 × 1 µm area, due to the pits and debris on the surface. Moreover, the specific surface area and the coefficient of friction, measured by Inverse Gas Chromatography (IGC), increased by 5 and 11%, respectively. Qian et al. (2010) also observed a slight enhancement of the nitrogen BET surface area of fibres, from 0.71 m^2^/g for oxidised IM7 carbon fibres to 1.71 m^2^/g after CNTs grafting (Figure 5c) [57]. Fibre surface roughness was qualitatively increased in the work of Hu et al. (2019) studying the spray coating of CNTs on carbon fibres (Figure 6a) for their incorporation in high-density polyethylene (HDPE) composites [58]. This change of fibre surface topography greatly enhanced the mechanical interlocking at the fibre/matrix interface, as shown on SEM images of fracture surfaces (Figure 6b,c). Indeed, the presence of CNTs coated on the surface of carbon fibres (Figure 6c) seems to increase the interfacial bonding between the HDPE matrix and the fibres, and also decrease voids in between, compared to pristine carbon fibres (Figure 6b).

The deposition of nano-objects on fibre surfaces can also modify their wettability, and hence ease their processing in composite materials. Zabihi et al. observed a better wettability towards epoxy resin for carbon fibres modified with APTES functionalized nanoclays. Indeed, contact angles between epoxy resin and carbon fibres measured by the drop-on-fibre technique decreased from ~42° to ~25° with functionalized nanoclays [50]. Using the same technique, Qian et al. found a similar trend with a decrease of the contact angle with PMMA matrix from 27.4° to 21.6° after the growth of CNTs on carbon fibre surfaces, indicating a better wettability of CNTs-carbon fibres towards the poly(methyl methacrylate) (PMMA) matrix [57].

The enhancement of the interfacial adhesion related to the presence of nano-objects on fibre surfaces can be determined by measuring the interfacial shear strength (IFSS) via different techniques. Single fibre fragmentation tests (SFFT) were conducted on hierarchically nanostructured carbon fibre/PMMA composites [57]. A positive impact of the CNTs deposition onto carbon fibres was highlighted with an increase of the IFSS from 12.5 MPa ± 0.2 to 15.8 MPa ± 0.4 compared to pristine carbon fibres. However, it should be noticed that the strength of CNTs modified carbon fibres dropped by ~15–17%, probably due, according to the authors, to the CVD reaction temperature of 750 °C used for CNTs deposition inducing the dissolution of iron catalyst in carbon fibres and lowering their mechanical properties [57]. On the other hand, Elhert and Sodano (2009) measured an IFSS improvement of 51% by SFFT, thanks to the functionalization of aramid fibres with ZnO nanowires imaged in Figure 5a compared to the aramid fibre/epoxy composite, without any degradation of the fibre tensile strength [56]. Finally, the deposition of nanoclays on carbon fibres also increased by 32% the IFSS with epoxy matrix [50]. Note that nanoclays were chemically functionalized by APTES, ensuring better chemical compatibility between the modified fibres and the matrix.

**Figure 7:**
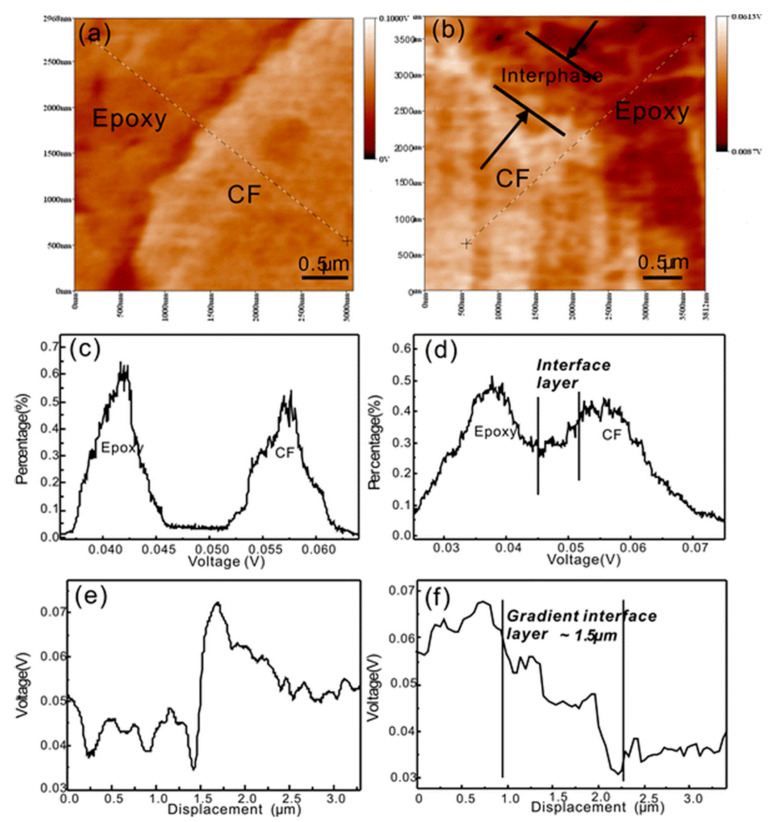
(**a**,**b**) Relative stiffness in the interphase region obtained by force modulation AFM, (**c**,**d**) probability histograms of relative stiffness and (**e**,**f**) stiffness change tendency with the appearance of a gradient interface layer between carbon fibres and epoxy resin for hierarchical carbon fibres modified with 1 wt% graphene nanosheets (**d**,**f**) (reprinted with permission from Chen et al., 2015 [21]).

Besides, it is important to point out that the deposition of nano-objects on fibre surfaces is likely to increase the local modulus of interphase regions and thus modifies the load transfer from the matrix to the fibres and among fibres within the composite structure. Chen et al. (2015) studied the deposition of graphene nanosheets on carbon fibres by liquid phase deposition (Figure 5d) for reinforcing epoxy-based composites [21]. Based on AFM measurements in force modulation mode (Figure 7a,b), the authors evidenced a gradual variation of the relative stiffness from the matrix to the fibres thanks to the presence of graphene nanosheets on carbon fibre surfaces.

The stiffness distribution, illustrated in Figure 7a,c, is split into two distinct zones corresponding to the carbon fibre and the epoxy matrix. This is completely modified by the presence of graphene on fibre surfaces with the appearance of a transition phase with local stiffening (Figure 7b,d). This gradient interface layer of about 1.5 µm was not observed with pristine carbon fibres (Figure 7e,f). A smoother transition in the interphase region, from the macroscale (carbon fibres) down to the nanoscale (macromolecular structure of the matrix), contributes to the reduction of local stress concentrations within the composite structure, allowing much more efficient stress transfer when the composite structure is under load. It should be noted that such AFM measurements require a meticulous preparation of the samples with a smooth and clean surface.

**Figure 8:**
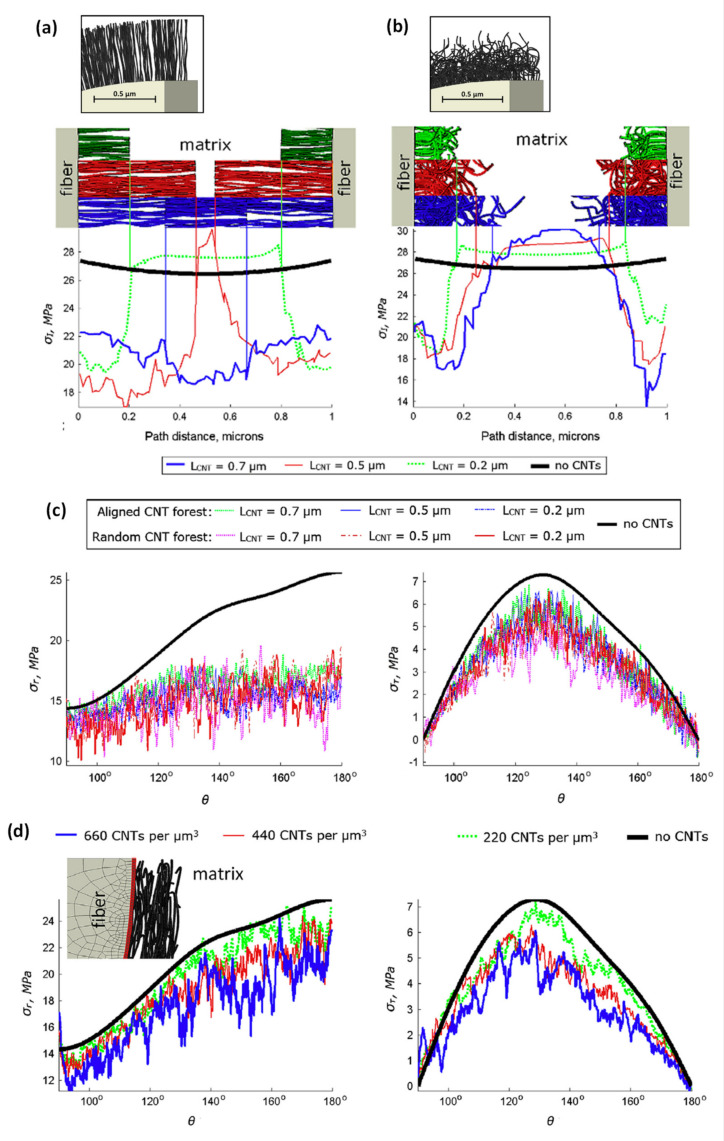
The maximum principal stress σ_I_ in the matrix along the shortest path between the fibres for (**a**) aligned and (**b**) random CNTs forests; (**c**) the effect of CNT grafting on normal interface stress σ_r_ and shear interface stress σ_τ_; (**d**) the effect of CNTs in the fibre coating on normal stress σ_r_ and shear stress σ_τ_ at the interface (reprinted with permission from Romanov et al., 2015 [59]).

To go further in the understanding of the effects of the presence of nano-objects at the interphase zone, Romanov et al. (2015) [59] modelled local stress distribution within CNTs modified unidirectional carbon fibre/epoxy composites, based on a 3D Finite Element model previously developed in [60]. The presence of CNTs on fibres is likely to change the mechanical performance of the interphase and especially to shift stress concentrations from the fibre interface to the end of CNT, modifying stress distribution within the material. The authors worked on different morphologies of deposited CNT on fibre, that is, wavy or quasi-straight, and deposition techniques with CNT growth from fibre surface or coating, inducing various orientations of CNT at the fibre/matrix interface, that is, radially aligned or randomly oriented (Figure 8). First, they found that CNTs caused sharp stress gradients in the matrix due to their high stiffness and aspect ratio. Moreover, the presence of CNTs generates stress concentrations at two scales: localised at the nanoscale around individual CNT, creating few micro-cracks, but also at the microscale taking into account CNTs collectively, implying more serious damages.

The authors focused on the analysis of the maximum principal stress in the matrix σ_I_, the shear/tangential and normal/radial stresses at the fibre/matrix interface, σ_τ_ and σ_r_ respectively, represented in Figure 8. First, the maximum principal stress σ_I_ was analysed in the matrix located between fibre surfaces (Figure 8a,b) for aligned and random CNT forests and showed contrasting results. For all configurations, the stress σ_I_ is much lower inside CNT forests (by 20–45%), compared to the reference without CNTs on the fibre surface, which displays an almost constant σ_I_ = 27 MPa (black curve). However, the stress in the resin-rich area is increased by 4–11% compared to the reference, depending strongly on the CNT length, due to the high stiffness and oriented nature of CNTs at the interface. Increasing the length L_CNT_ to 0.5 µm induces a narrower CNT-free matrix region and so higher stress (Figure 8a, red curve). Contrarily, the stress concentration between fibres is drastically reduced in the case of overlapping CNT forests observed at L_CNT_ = 0.7 µm (Figure 8a, blue curve). Moreover, in the area where CNT forests are interpenetrated, an additional loss of stress is observed. However, this overall stress decrease at the microscale can cause damages in other areas. Indeed, CNT-free matrix zones in CNT forests are narrower, which induces an increase of the nano-scale stress concentrations in these matrix areas.

Considering Figure 8c,d, it seems that the CNT orientation on fibre surfaces, which can be controlled by the deposition technique (growth or coating), induces different behaviours considering radial or tangential stresses. First, both random and aligned CNT forests growing on fibre reduce overall stress at the fibre/matrix interface with a decrease of the normal stress up to 40% and in the shear stress up to 29%, compared to the reference composite (Figure 8c). On the other hand, CNTs coated on the fibre surface induce a lower decrease in the normal stress up to 22% but a greater decrease in the shear stress up to 36% (Figure 8d). This result can be explained by the orientation of coated CNTs, which are aligned quasi-parallel to the fibre surface and so quasi-perpendicular to the normal stress direction. It can be assimilated to the loading of a quasi-unidirectional composite in the transverse direction, and so dominated by the matrix behaviour. To conclude, the deposition technique of CNTs on fibre (growth or coating) implies different CNT orientations, that is, quasi-radial or quasi-parallel to the fibre, which is likely to influence greatly the stress distribution and the magnitude of the local normal and shear stresses.

Finally, as observed in biological systems, nanoparticles can hinder crack propagation within the composite structure, which then follows a zig-zag path preferentially at interfaces hence inducing many crack deflections [19]. In this regard, Zabihi et al. [50] observed that the deposition of nanoclays on carbon fibres favoured the deviation of cracks along the fibre/matrix interfaces. Indeed, failure surfaces revealed the presence of big debris of epoxy resin attached to the hierarchical carbon fibres modified with nanoclays, indicating a more tortuous crack propagation favouring energy dissipation during composite failure. This change in failure mechanism enhanced fracture toughness of composite materials.

The hierarchical structuration of fibres can thus modify their surface topography and wettability towards polymer matrices and is likely to enhance interfacial adhesion, stress transfer and distribution within the composite structure while also modifying failure mechanisms. As a result, increased strength and fracture toughness can be achieved. All data and observations are synthesized in Table 1. These effects are strongly related to the dimensions and physical properties of the nano-objects and their orientation and interactions with fibre surfaces. Based on this concept, several research works focused on the development of hybrid hierarchical composites (Figure 4b) reinforced with either synthetic or natural fibres modified by synthetic/mineral nanoparticles such as ZnO, CNTs, or bio-based nanoparticles such as nanocelluloses (cellulose nanocrystals, bacterial cellulose).

### 3.2. Hybrid Hierarchical Fibre Reinforced Composites

Idumah and Hassan (2016) published a review on the development of hybrid assemblies in polymer nano-biocomposites [25]. The reviewed works were mainly focused on hybrid composite systems incorporating nanofillers within the bulk of the polymer matrix [61,62,63], and only a few examples dealt with the implementation of hierarchical fibres to tailor the interphase in composites. This section will be focused on the development of hierarchical composites modified by nanoparticles located on fibre surfaces, hence following the concept of composites reinforced with hierarchical fibres.

#### 3.2.1. Hierarchical Natural Fibres Modified by Synthetic/Mineral Nanoparticles

**Figure 9:**
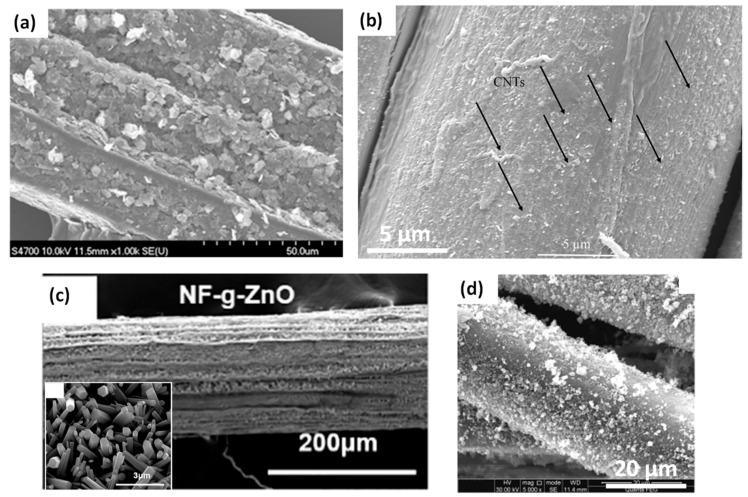
SEM images of hierarchical natural fibres (**a**) 5 wt% of graphite nanoplatelets (xGnP) coated on kenaf fibres, (**b**) 1 wt% carboxyl-functionalized CNTs (COOH-CNTs) coated on flax fibre yarns, (**c**) laterally-grown ZnO nanowires on sisal fibres, (**d**) 2.34 wt% grafted nano-TiO_2_ on flax fibres of yarns (reprinted with permission from Han et al., 2012 [64], Li et al., 2015 [65], Yang et al., 2020 [66] and Wang et al., 2015 [67]).

Different systems of synthetic or mineral nanoparticles deposited on natural fibres were developed in the literature, primarily to enhance the mechanical properties of composites. The morphologies of some of these hierarchical natural fibres are illustrated in Figure 9.

Han et al. (2012) studied the coating of kenaf fibre bundles by exfoliated graphite nanoplatelets (xGnP) to reinforce poly (lactic acid) (PLA) based composites [64]. This coating was achieved by simple physical adsorption into acetone solvent with no chemical bonding between xGnP and kenaf bundles (Figure 9a). The authors tested different amounts of xGnP (0, 1, 3, and 5 wt%) dispersed in the solution and observed, via thermogravimetric analysis, a saturation effect of coated xGnP on fibres, inducing the dispersion of the xGnP in excess within the PLA matrix during the compounding process. A decrease of the composite flexural strength was observed with the addition of xGnP, suggesting a weaker interface in the xGnP kenaf fibres/PLA composite. The authors explained these results by the hydrophobic character of xGnP nanoparticles, hence less compatible with the more hydrophilic PLA matrix, implying a decrease of the interfacial adhesion. However, still according to the authors, xGnP deposited on kenaf fibre surfaces could provide better fibre dispersion within PLA by reducing attractive interactions among kenaf fibres. Besides, the flexural modulus of the composite was increased by more than 165% for PLA composite reinforced with 40 wt% kenaf fibres modified with 5 wt% of xGnP. The authors explained this result by the synergistic effect of both reinforcements (kenaf fibres and xGnP). Indeed, they measured an enhancement of PLA modulus by ~25% with the addition of 5 wt% xGnP and by ~110% with the addition of 40 wt% kenaf fibres.

**Figure 10:**
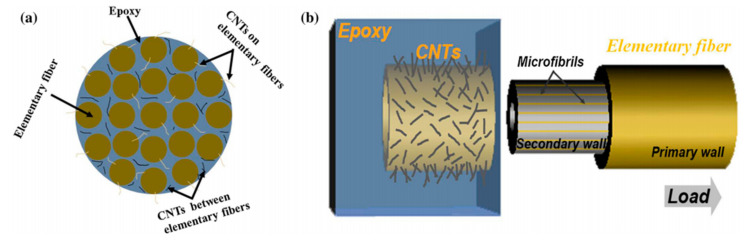
(**a**) Distribution of CNTs in flax yarn reinforced epoxy composites and (**b**) interlocking mechanism and failure process of composites (reprinted with permission from Li et al., 2015 [65]).

Li et al. (2015) studied the coating of carboxyl-functionalized CNTs (COOH-CNTs) on the surface of flax yarns by the spray-drying process (0.5, 1, and 2 wt% COOH-CNTs suspension concentrations; drying 120 °C for 8 h) and its effect on mechanical properties of flax fibre reinforced epoxy composites [65]. The flax yarn coated with 1 wt% COOH-CNTs in Figure 9b shows uniformly dispersed and randomly oriented COOH-CNTs on the fibre surfaces, linked by hydrogen bonds with available hydroxyl groups of flax fibres. They observed an increase of the IFSS between flax yarns and epoxy resin by 26% thanks to the coating of 1 wt% COOH-CNTs on fibres. The interlaminar shear strength (ILSS) of the prepared composites was also increased by 20% for the coating of 1 wt% COOH-CNTs. The complex interfacial region, illustrated in Figure 10a, shows COOH-CNTs coated on flax yarns by hydrogen bonds but also COOH-CNTs located between elementary fibres inside the yarn. According to the authors, several types of interfaces must be considered in COOH-CNTs-coated flax yarn/epoxy composites such as fibre/matrix interfaces but also those between cell walls within elementary flax fibres. Figure 10b illustrates the interlocking mechanism with the epoxy resin brought by the presence of COOH-CNTs coated on flax fibres, which induces an enhancement of the load transfer in the interphase zone.

The deposition or growth of ZnO nanowires on natural fibres has been investigated in some studies. Yang et al. (2020) modified sisal fibres with laterally grown ZnO nanowires to enhance the interface in poly (lactic acid) (PLA)/sisal biocomposites [66]. The deposition method was based on a two-step hydrothermal method with (i) dip-coating or immersion of sisal fibres in a seed suspension of ZnO colloidal particles and (ii) growth of unidirectional ZnO crystals from the seeded surface of sisal fibres (Figure 9c). The estimated amounts of coated ZnO nanowires on sisal fibres were 1.8 wt%, 8.4 wt%, and 16.7 wt% for respectively 1, 2, and 6 dip-coating cycles. The ZnO nanowires modified sisal fibres provided excellent interfacial adhesion with the PLA matrix, attested by an increase of IFSS and debonding energy of 157% and 400%, respectively, for the PLA/sisal fibres modified with 6 dip-coating cycles as compared to the PLA/pristine sisal fibre biocomposites. However, these strong increases of IFSS and debonding energy values between neat sisal fibre and PLA matrix measured by the pull-out technique (IFSS of 2.8 MPa to 7.2 MPa) must be taken with caution. Indeed, these values are very low compared to those found in the literature, that is, Taha et al. (2010) obtained an IFSS value of 17.1 MPa without any fibre surface treatment [68]. This hierarchical structure at the surface of sisal fibres improved the mechanical interlocking fibre/matrix, suggesting a more efficient load transfer at the interface. The authors modelled the stress distribution along the fibre/matrix interface during a single-fibre pull-out test with or without ZnO nanowires on sisal fibres. The stress generated at the interface was small and only a part of the interface worked efficiently, because of the weak interfacial cohesion between pristine sisal fibre and PLA matrix. In contrast, the laterally-grown ZnO nanowires on sisal fibres contributed to the anchoring of nanowires in the PLA matrix with a high mechanical interlocking. Consequently, ZnO coated sisal fibres resulted in enhanced interfacial adhesion with a more efficient transfer of loads within the composite.

Sbardella et al. (2021) studied the growth of ZnO nanorods on flax yarn surface in order to improve the interfacial adhesion in flax/epoxy matrix biocomposites [69]. The hydrothermal treatment was provided to obtain highly oriented ZnO nanostructures, that is, hexagonal wurtzite nanorods. This treatment can be divided into two main steps, (i) the seeding and (ii) the epitaxial growth of ZnO nanoparticles, and was optimized in this work by testing different parameters, such as the number of seeding cycles (1 to 3 cycles), the growth time (1 to 5 h) or the refresh of the treatment solution. First, the authors obtained the higher aspect ratio for ZnO nanorods with 1 seeding cycle and 5 h of growth time: L/d = 14.6 (L = 3.0 µm ± 0.1; d = 0.20 µm ± 0.03) against L/d = 4 after 2 h of growth time. Moreover, they showed that this treatment does not affect the flax yarn’s tensile strength. Finally, they measured the IFSS by SFFT between ZnO 5 h_2.5 modified flax yarn (1 seed cycle, 5 h, one refresh of the treatment solution at 2.5 h) and the epoxy matrix. They found a lower IFSS of 12.7 MPa ± 2.4 compared to 19.3 MPa ± 3.7 for the neat flax yarn. The authors explained this loss of interfacial adhesion result by the heterogeneity of flax yarn diameters, which could negatively affect SFFT data. Moreover, they observed an increase in diameter with the ZnO layer on yarn surface with ~340 µm compared to ~250 µm for the neat flax yarn, and possible swelling and untwisting during the hydrothermal treatment, which can involve difficulties in samples comparison. It can be noticed that these IFSS values measured on flax/epoxy systems by the SFFT technique are very different from those obtained by Li et al. based on the pull-out technique [65], that is, 43.7 MPa to 55.0 MPa. This difference could be ascribed to the use of different techniques. Besides, the study at the scale of fibre yarns could be more difficult to interpret due to their complex structure as compared to elementary fibres. Nevertheless, the work of Sbardella et al. gives more data on the testing parameters used for the SFFT test (reproducibility, crosshead speed, load cell, etc.).

**Figure 11:**
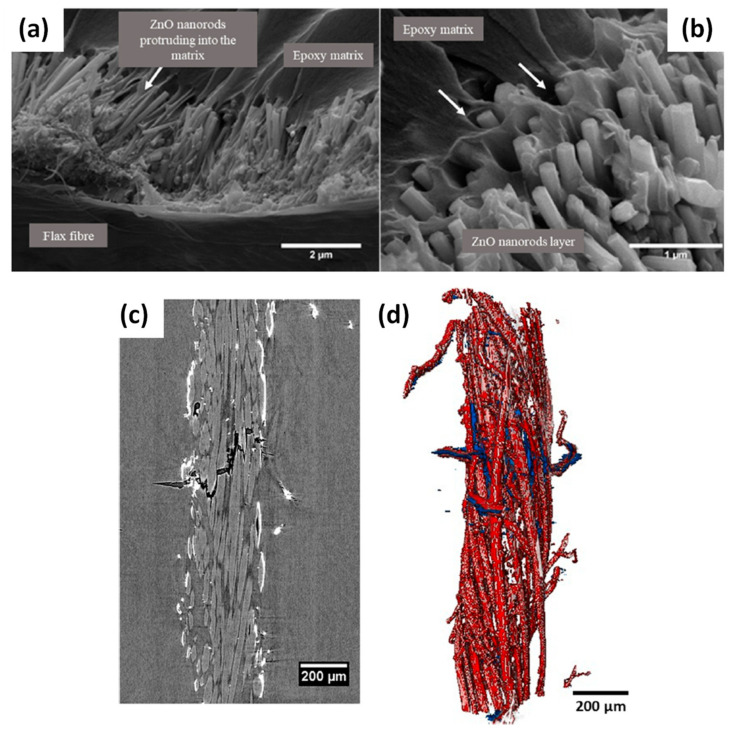
(**a**,**b**) FE-SEM micrographs showing fracture surfaces for ZnO-flax/epoxy single yarn composites. Micro-computed tomography observation for the ZnO-modified flax yarn/epoxy matrix with (**c**) high-resolution micro-CT image of the flax embedded in the epoxy resin and (**d**) volumetric reconstruction of the flax yarn and the fracture zone: the flax yarn is represented in dark grey, the fracture zone in blue and the ZnO coating in red (reprinted with permission from Sbardella et al., 2021 [69]).

To complete the analysis on the interfacial adhesion, the authors observed fracture surfaces of the ZnO 5 h_2.5 modified flax yarn/epoxy SFFT sample as shown in Figure 11a,b. It is clearly obvious that the vertically aligned ZnO nanorods on the flax yarn surface are interconnected with the epoxy matrix, creating a bridge between the yarn and the matrix and probably an enhanced mechanical interlocking. The high-resolution microtomography analysis showed homogeneous ZnO surface coating on flax yarn (Figure 11c), bright areas, without penetration within the yarn. Moreover, the 3D reconstruction of the fracture region along the flax yarn (Figure 11d) shows the presence of matrix cracks and a debonding between yarns and the epoxy matrix to be very limited, supporting that the interfacial adhesion was enhanced in the biocomposite.

Ovalle-Serrano et al. (2015) also studied the deposition of ZnO nanowires on natural fibres, especially fique fibres from Columbia. They investigated another strategy based on a co-precipitation method with the use of a precursor ZnSO_4_, NaOH for the hydroxide formation and thermal/ultrasound energy [70]. However, the authors did not use the modified fique fibres for the manufacturing of biocomposite materials.

**Figure 12:**
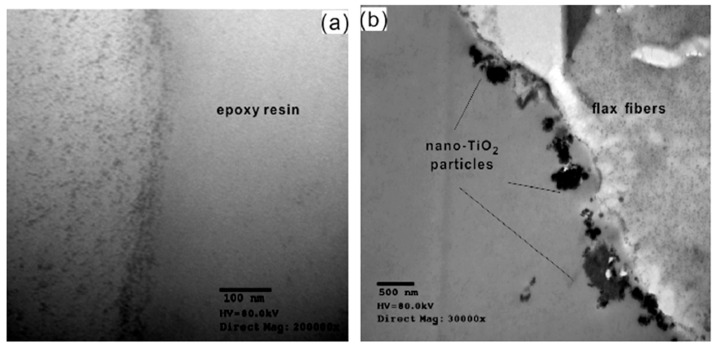
Transmission Electron Microscope (TEM) images of flax fibre cross sections: (**a**) control flax fibre, (**b**) a nano-TiO_2_ grafted flax fibre (reprinted with the permission from Wang et al., 2015 [67]).

Wang et al. (2015) modified flax yarns by the grafting of nano-TiO_2_ particles (Figure 9d) and studied the effects on tensile and bonding properties of elementary fibres and unidirectional fabric reinforced epoxy composite plates [67]. They cleaned (ultrasonic bath 80 W, 20 °C, 6 h) and dried (20 °C, 24 h) flax fibres before their dipping in nano-TiO_2_/KH560 during 15 min under sonication and then washed grafted fibres with ethanol and distilled water for 1h. They also performed NaOH and silane coupling agent treatments to compare different types of flax fibre modifications. They tested different suspension concentrations and succeeded to graft from 0.89 wt% up to 7.14 wt% nano-TiO_2_ on flax fibres (estimations based on XPS measurements). Figure 12b shows the presence of nano-TiO_2_ particles at the surface of flax fibres compared to pristine flax fibres (Figure 12a). However, nano-TiO_2_ particles are not uniformly distributed on flax fibres’ surfaces with the presence of aggregates (150 nm to 300 nm).

The optimum nano-TiO_2_ grafting content on flax fabrics was 2.34 wt% and contributed to the increase of tensile strength of flax fibres by 23.1%. Moreover, the IFSS between flax fibres and the epoxy resin was improved by 40.5%. In comparison, the modification of flax fibres with a silane coupling agent (KH-560) showed a lower increase of the IFSS with 23.4%. Flexural strength of flax fibre reinforced epoxy composite plates was increased by 31.4% when grafting 2.34 wt% of nano-TiO_2_, by 19% for the silane treatment and by 9.5% for NaOH treatment, compared to the untreated flax fabric/epoxy composite. These results suggest that the creation of hierarchical flax fibres with the presence of nano-TiO_2_ induces a more significant improvement of the ultimate mechanical properties of flax/epoxy composites (tensile and flexural tests) than chemical treatments such as NaOH or the functionalization with a silane coupling agent.

**Figure 13:**
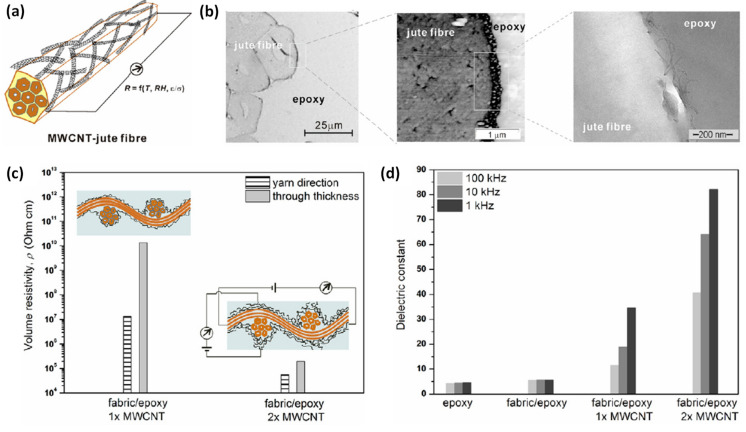
(**a**) Schematic presentation of single multi-walled CNT modified jute fibre, (**b**) from left to right are the SEM, EFM phase, and TEM images of the cross-section of the MWCNT-jute fibre/epoxy biocomposite, (**c**) comparison of the volume electrical resistivity of jute fabric/epoxy composites along yarn and through thickness directions, and (**d**) dependence of dielectric constant of jute fabric/epoxy composites on the measurement frequency and fabric treatment conditions (reprinted with permission from Zhuang et al., 2011 [71]).

Finally, the work of Zhuang et al. (2011) shows the possibility to develop hybrid hierarchically nanostructured jute fibres and jute/epoxy composites displaying multi-functional properties [71]. Jute fibres and fabrics were treated in acetone and then hot water in a sonication bath prior to the dip-coating (one hour) of multi-walled carbon nanotubes (MWCNTs) on fibre surfaces, then air-dried overnight in a fume hood and under vacuum (40 °C, 8 h). Jute/epoxy composites were prepared by a vacuum-assisted process with 20.5 wt% fibre content. The authors observed the formation of an electrically semiconducting MWCNT network on jute fibre surfaces, illustrated in Figure 13a, with a thickness of around 100 nm after one dip-coating step (Figure 13b).

The authors pointed out that the surface properties of jute fibres, functionalized with multi-walled carbon nanotubes, modified the electrical conductivity of the composites. Moreover, the content and extent of conductive pathways could be easily controlled considering the jute fabric treatment conditions. Indeed, volume electrical resistivity measurements showed that two MWCNT dip-coatings of jute fabrics provided composites with two orders of magnitude lower volume resistivity values, and also conductive pathways along yarn direction and even in their thickness (Figure 13c). The study of the frequency-dependent dielectric properties of jute fabric/epoxy composites showed a significant increase of the dielectric constant with the number of MWCNTs dip-coating (Figure 13d). Finally, the results evidenced the potential of MWCNTs-jute fabric/epoxy hybrid hierarchical composites as temperature, relative humidity, or strain/stress sensors.

#### 3.2.2. Hierarchical Synthetic Fibres Modified by Bio-Based Nanoparticles

Synthetic fibres have been modified with different types of cellulose-based nanoparticles. Nanocelluloses, that is, cellulose nanofibrils (CNF) and cellulose nanocrystals (CNC), are mainly obtained by an extraction procedure from lignocellulosic biomass [45]. CNF are long and flexible (length: few micrometres; width: 5–100 nm), semi-crystalline with a lateral nanometric dimension, mainly influenced by pre-treatments (mechanical, chemical or enzymatic) with aspect ratio L/d from 10 to 100 and Young’s modulus around 81–98 GPa [72,73]. CNC are rod-shaped stiff nanoparticles (length: 100 nm–few micrometres; width: five to several tens of nanometers) displaying high crystallinity, providing a high specific surface area up to 150–800 m^2^/g [45,73] and a high Young’s modulus around 100–130 GPa [74]. Hydrolysis of cellulose by sulfuric acid is the most common way to produce CNC, which induces the presence of half sulfate ester groups (–OSO_3_^−^) on their surface. Bacterial cellulose (BC) displays a lateral dimension around 25–100 nm and a length of several micrometres [75]. BC is produced by the bacteria *Acetobacter xylinum*, which synthesizes cellulose nanofibrils from low molecular weight compounds (sugars, alcohol, etc.) [76]. This fermentation process requires the accurate control of different bioprocess parameters such as culture medium, temperature, incubation time, pH. An important difference, compared to CNF and CNC extracted from lignocellulosic biomass, is that BC is devoid of hemicellulose and lignin. BC nanofibres display high crystallinity (up to 84–89%) and Young’s modulus of roughly 78 GPa [77,78].

**Figure 14:**
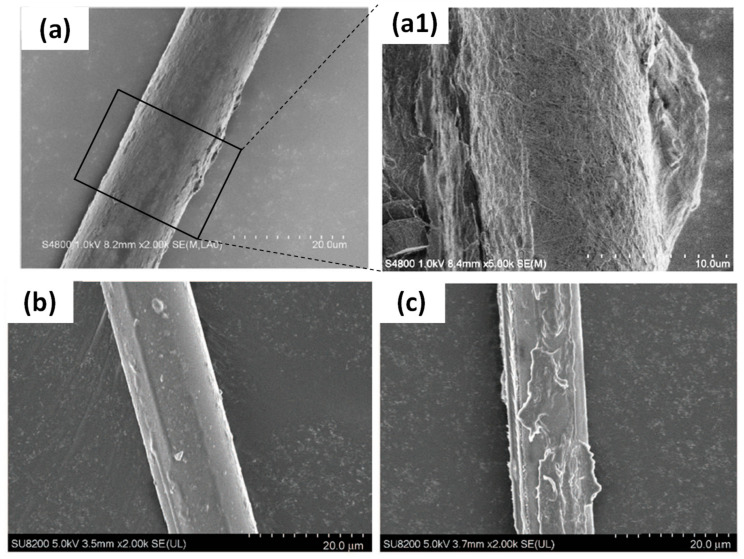
SEM images of hierarchical glass fibres (**a**) and (**a1**) BC grown on glass fibres, (**b**,**c**) CNC coated glass fibres with respectively 1 wt% and 5 wt% CNC content in suspensions (reprinted with permission from Chen et al., 2014 [79] and Asadi et al., 2016 [80]).

The following examples of hybrid hierarchically nanostructured fibre reinforced composites are composed of synthetic fibres, more especially glass fibres, coated with bio-based nanoparticles such as BC or CNC (Figure 14).

**Figure 15:**
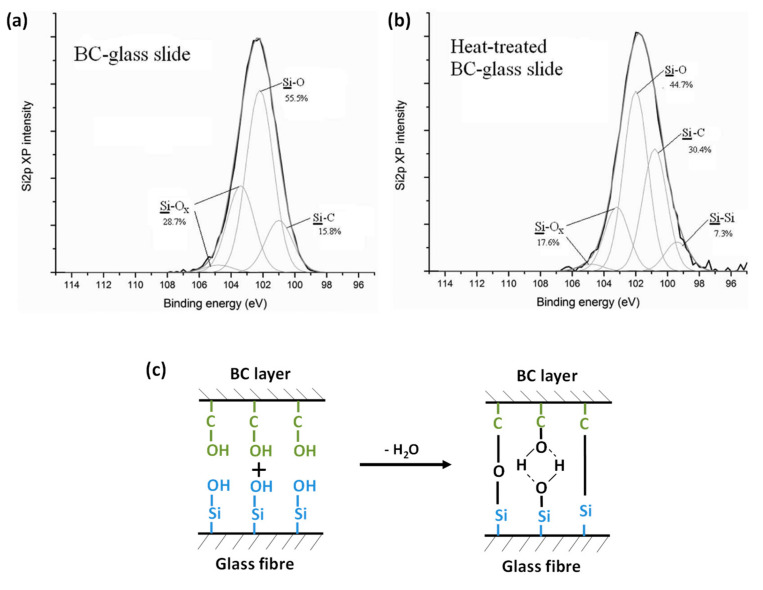
(**a**,**b**) Si 2p XP spectra of BC-glass slide before and after heat treatment at 140 °C during 24 h, (**c**) Schematic representation of chemical bonding between hydroxyl groups of bacterial cellulose (BC) and glass fibres and possible formation of C-Si and C-O-Si covalent bonds (reprinted with permission from Chen et al., 2014 [79]).

Chen et al. (2014) studied the influence of the dipping time of glass fibres in the BC culture medium and the effect of heating post-treatment of BC deposited fibres on the final interfacial properties between glass fibres and epoxy resin [79]. BC was deposited on glass fibres by fermentation at 30 °C for several hours in a culture medium (Figure 14a(a1)). First, they obtained by microbond test the optimum IFSS value for 1 h dipping time with an increase from 14.1 MPa ± 1.9 to 21.5 MPa ± 2.2 compared to the untreated glass fibre/epoxy system. Moreover, it appears that the heating post-treatment of BC-glass fibres at 140 °C during 24 h increased slightly the IFSS by 3%. Moreover, the authors explained that this heating post-treatment could enhance adhesion between BC and glass surfaces. Indeed, they conducted XPS measurements on the surface of unheated and heat-treated BC-glass slides (140 °C, 24 h). The surface chemical composition of both samples was shown in high-resolution Si 2p XP spectra (Figure 15a,b). It is obvious that after heating of BC-glass slide, Si-C bonds increased from 15.8% to 30.4%, formed by the reaction between Si-OH of glass fibres and C-OH of BC and so contributing to better BC/glass adhesion. The optimization of treatment parameters increased the BC-glass fibre adhesion with the creation of hydrogen bonds after 1 h dipping and led to stronger bonding between glass fibres and BC, that is, C-Si and C-O-Si covalent bonds, after heating post-treatment at 140 °C during 24 h, as illustrated in Figure 15c.

**Figure 16:**
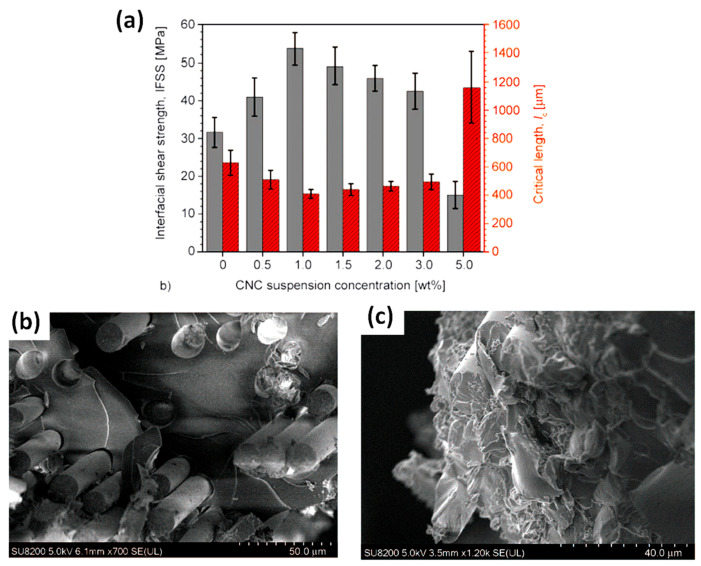
(**a**) SFFT results with the IFSS (solid grey bars) and critical fragmentation length (striped red bars) for short glass fibres/epoxy composites as a function of CNC concentration in suspension, failure surfaces observed for (**b**) 30 wt% uncoated glass fibre and (**c**) 30 wt% CNC-coated glass fibre (1 wt% CNC in suspension) reinforced epoxy composites (reprinted with permission from Asadi et al. (2016) [80]).

Asadi et al. (2016) prepared short glass fibre/epoxy composites with pristine and CNC-treated glass fibres (Figure 14b,c) by the immersion/dipping of chopped glass fibre roving (around 25 mm length) in CNC aqueous suspensions between 0 and 5 wt% CNC contents [80]. First, they observed that favourable interactions between hydroxyl groups of CNC and glass fibres promote physical adsorption of CNC at the surface of the roving but also the partial penetration of CNC within the roving, resulting in heterogeneous coating of individual glass fibres. The interfacial shear strength (IFSS) measured by SFFT showed an optimum value for 1 wt% CNC in suspension with an increase of ~69% compared to the untreated glass fibre/epoxy composite (Figure 16a).

The authors assumed that too much CNC on fibre surfaces could induce slippages between CNC layers, decreasing the load transfer efficiency across the interphase. Moreover, the composite reinforced with CNC-modified glass fibres prepared in a 2 wt% suspension showed a decrease of the tensile strength of ~12%, while the IFSS measured on the same system was higher than for pristine glass fibres. The authors attributed this antagonism to various mechanisms and in particular to the incomplete impregnation of the epoxy resin around and within the glass roving coated by a thick CNC layer, possibly resulting in the formation of voids that could decrease the composite’s strength. SEM images of failure surfaces are presented in Figure 16b,c. The fibre/matrix interfacial adhesion appears very weak for the untreated glass fibre/epoxy composite, with mainly interfacial debonding and fibre pull-outs, characteristic of an adhesive interfacial failure. In contrast, the presence of CNC on the surface of glass fibres results in matrix cracking and fibre breakage, and hence more cohesive interfacial failure.

Combination of nanoparticles was also tested with the deposition of both cellulose nanocrystals and carbon nanotubes on the surface of carbon fibres, in order to enhance the interlaminar shear strength (ILSS) in composite laminates [81,82]. In this hybrid treatment, CNCs are used as a dispersing and stabilizing agent of CNTs in water before the treatment of carbon fibres. The results obtained concerning the mechanical properties of composites are promising with an increase of the flexural strength and the ILSS by 33% and 35%, respectively when treating carbon fibres with 0.2 wt% CNC 0.2 wt% CNT in suspension. This review on hybrid hierarchically nanostructured fibre reinforced composites highlights the challenge of depositing nano-objects that can have no physico-chemical affinities with the reinforcing fibres used. All data and observations are synthesized in Table 2.

Indeed, the deposition or growth of CNT, ZnO, or graphene requires in some cases very high temperatures, not compatible with natural fibres. While the preparation of hierarchical synthetic fibres such as carbon fibres is often carried out by the CVD method (750 °C for 1 h, C_2_H_2_ carrier gas [57]), the deposition of synthetic nano-objects for the creation of hierarchical natural fibres requires gentler techniques such as hydrothermal deposition, spray-drying or dipping in suspension. It should also be noted that the growth of synthetic nano-objects on the surface of fibres has been transposed to biobased nano-objects by the use of bacterial cellulose, growing directly on the fibres by incubation.

Most of the works on the development of synthetic and hybrid hierarchical fibre reinforced composites evidenced a reinforcement of the interphase zone thanks to hierarchical fibres, with improved interfacial adhesion measured by different techniques such as IFSS, ILSS or flexural tensile tests. The next challenge is to transpose this bio-inspired concept of hierarchical interphase to fully bio-based reinforcements, that is, natural fibres modified by bio-based nanoparticles. The following section will focus on hierarchical biocomposites as schematized in Figure 4c, in particular the deposition of cellulose-based nanoparticles on various natural fibres.

## 4. Hierarchical Interphase in Natural Fibre Reinforced Biocomposites

The main advantage when treating natural fibres with cellulose-based nanoparticles (i.e., BC, CNC, CNF) is their spontaneous adsorption on fibre surfaces. Indeed, they have a mutual affinity brought by hydrogen bonding and van der Waals interactions. Moreover, surface modification can be performed directly in water without the use of organic solvents or prior fibre pre-treatments that would be less eco-friendly and could induce fibre degradation [83].

### 4.1. Hierarchical Natural Fibres Modified by Bacterial Cellulose

**Figure 17:**
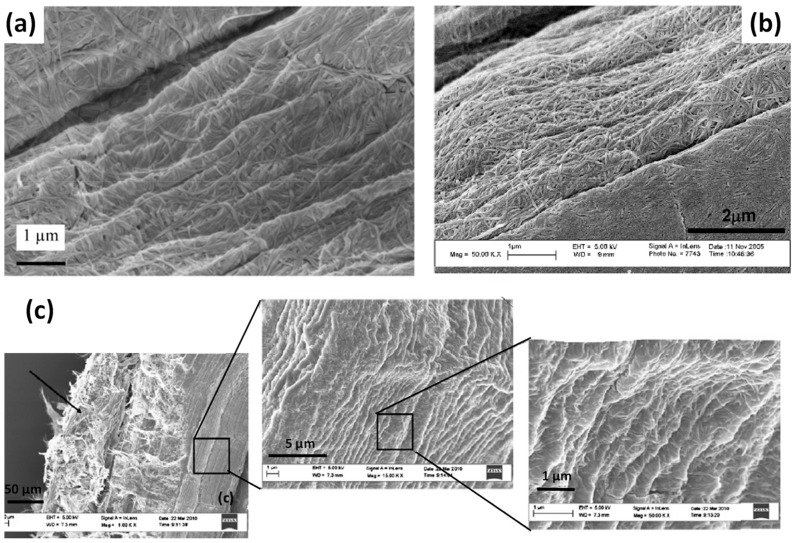
SEM images of (**a**) hemp fibres coated by BC, (**b**) sisal fibres coated by BC and (**c**) “hairy” short sisal fibres coated by BC (reprinted with permission from Pommet et al., 2008 [28], Juntaro et al., 2007 [84] and Lee et al., 2012 [86]).

Several works report on the deposition of BC on natural fibres to create hierarchical fibres with the aim of enhancing the fibre/matrix interface in biocomposites. For example, BC was grown on the surface of sisal and hemp fibre bundles (7 days, 30 °C) for their further incorporation in bio-based matrices, that is, PLA and cellulose acetate butyrate (CAB) [28,84,85]. The authors performed SEM observations of BC grafted-hemp fibres (Figure 17a) and BC grafted-sisal fibres (Figure 17b) and found that BC nanofibres were randomly oriented and covered fibre surfaces almost completely.

The biocomposite showing the most promising results was PLA/BC-grafted sisal fibres with an increase of longitudinal and transverse tensile strengths by 44% and 68%, respectively, compared to the untreated sisal fibre reinforced PLA biocomposite. The IFSS measurements showed a positive effect of the BC treatment on the sisal/PLA matrix interfacial adhesion with an improvement from 12.1 ± 0.5 MPa to 14.6 ± 1.2 MPa. In the case of BC modified hemp fibres, the authors observed “glued” elementary fibres forming bundles, which could hamper good impregnation with the matrix during manufacturing, and create defects and voids within the biocomposite microstructure. This is likely to be at the origin of the bad or unchanged results observed for hemp fibre reinforced biocomposites with both PLA and CAB matrices. Moreover, the better mechanical properties (strength and stiffness) obtained for PLA based biocomposites compared to CAB based ones could be explained, according to the authors, by the polar functional groups of PLA likely to form hydrogen bonds with BC hydroxyl groups present at the fibre surface, hence providing a better fibre/matrix interfacial adhesion.

**Figure 18:**
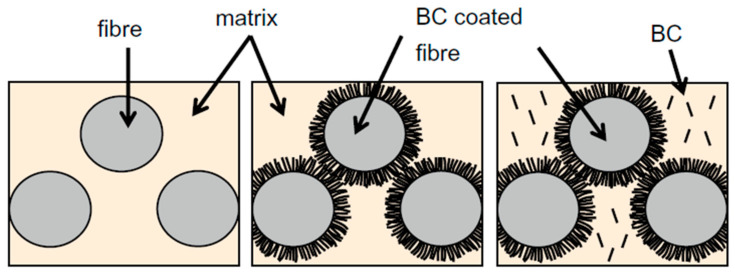
Illustration of the different hierarchical sisal fibres/PLA biocomposites with or without the addition of bacterial cellulose (BC). (**Left**) with untreated sisal fibres; (**middle**) with BC coated sisal fibres and (**right**) with BC coated sisal fibres and BC in the matrix bulk (reprinted from Lee et al., 2012 [86]).

Lee et al. (2012) also prepared BC-coated sisal fibres by dipping the sisal fibres in an aqueous dispersion of freeze-dried BC (3 days, room temperature) [86]. Based on two different drying processes, they obtained “dense” and “hairy” BC coated sisal fibres. The resulting “hairy” BC fibres are illustrated in Figure 17c with BC nanofibres oriented perpendicular to the fibre surface. The loading content of BC on sisal fibre was around 10 ± 1 wt%. The specific surface area of sisal fibres, measured by nitrogen adsorption/desorption isotherms, was slightly increased from 0.10 m^2^/g ± 0.01 to 0.77 m^2^/g ± 0.03 and 0.49 m^2^/g ± 0.03 for respectively “dense” and “hairy” BC coated sisal fibres. According to the authors, the lower specific surface area of “hairy” BC-sisal fibres might be due to the agglomeration of BC nanofibrils on the fibres when they are pressed between filter papers, reducing the accessible area for nitrogen adsorption. Then, sisal fibre/PLA biocomposites were prepared with untreated and BC-coated sisal fibres but also with the dispersion of freeze-dried BC in the bulk of the PLA matrix. The different BC/sisal fibres/PLA systems are shown in Figure 18.

**Figure 19:**
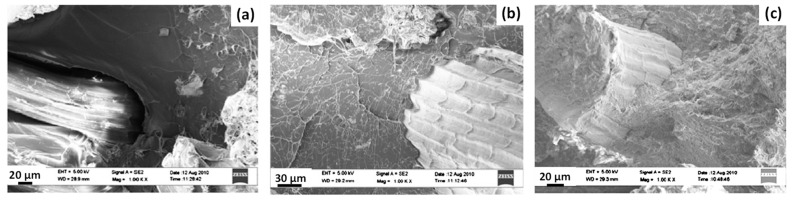
Fractured surfaces of hierarchical BC/sisal fibres reinforced PLA biocomposites, (**a**) untreated sisal fibres, (**b**) “hairy” BC-coated sisal fibres and (**c**) “hairy” BC-coated sisal fibres with BC dispersed in PLA matrix (reprinted with permission from Lee et al., 2012 [86]).

Considering the tensile performances of the different sisal fibre/PLA biocomposites, the combination of BC-coated sisal fibres with also BC dispersed in PLA seems to give the best results whatever the drying process. Indeed, tensile tests highlighted an increase in Young’s modulus and tensile strength by 27% and 16% respectively, for PLA/“dense” BC-coated sisal fibres and by 24% and 18% respectively, for PLA/“hairy” BC-coated sisal fibres, both containing BC in the PLA matrix, compared to PLA/untreated sisal fibre biocomposite without added BC. According to the authors, these results could be explained by a possible stiffening of the PLA matrix with the presence of BC and additional interactions such as hydrogen bonds and van der Waals interactions between BC dispersed in the matrix and BC coated on the surface of sisal fibres. Indeed, SEM images of fractured surfaces of biocomposites (Figure 19) highlighted a more cohesive interface between sisal fibres and PLA matrix for “hairy” BC-coated fibres. This was even more obvious with “hairy” BC-coated fibres and BC dispersed in the matrix. Indeed, bonded PLA matrix was observed on sisal fibres (Figure 19c), hence favouring matrix and fibre breakage rather than fibre debonding.

**Figure 20:**
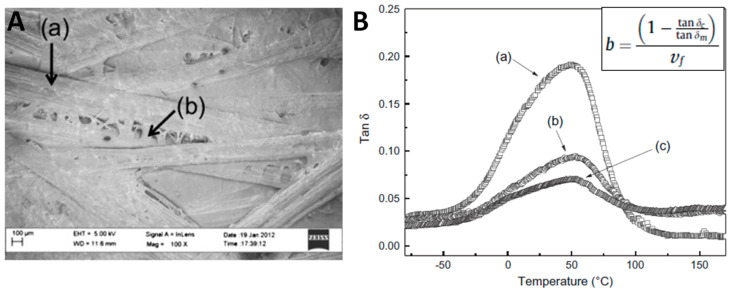
(**A**) SEM micrograph of sisal fibre with (**a**) preform using (**b**) BC as binder; (**B**) results of DMTA tests with tan δ as a function of temperature for (**a**) neat polyAESAO, (**b**) sisal-polyAESO and (**c**) BC-sisal-polyAESO biocomposites (reprinted with permission from Lee et al. (2012) [87]).

Lee et al. (2012) also investigated the manufacturing of a non-woven sisal preform treated with bacterial cellulose for reinforcing acrylated epoxidised soybean oil (AESO) resin [87]. Sisal fibre bundles (10 mm length) were dipped in a suspension of BC overnight and pressed to obtain the non-woven sisal preform bound by BC as illustrated in Figure 20A. The resulting sisal preform strongly increased both the tensile and flexural Young’s modulus of the AESO-based biocomposite, by 75% and 142% respectively, and tensile and flexural strength by 71% and 116% respectively, compared to the untreated sisal preform/AESO biocomposite. Dynamic mechanical thermal analysis (DMTA) was performed on biocomposites and results are represented in Figure 20B. The damping factor *tan δ* ($\tan\delta =E^{\prime \prime }/E^{\prime }$) is likely to be affected by the molecular mobility of macromolecular chains within the matrix and at the fibre/matrix interphase [88,89]. For sisal preform/AESO biocomposites [87], the DMTA curves showed a decrease of the damping factor from 0.09 to 0.07 due to the presence of BC on sisal fibres, characteristic of improved fibre/matrix interactions and restricted molecular motions at the interface. The fibre/matrix interfacial strength indicator *b* was calculated (see relationship in Figure 20B, with *tan δ_c_* and *tan δ_m_* the damping factors of the composite and the neat matrix respectively; *V_f_* the fibre volume fraction). A higher *b* value was obtained for the BC-sisal preform/AESO biocomposite, highlighting an increase of the interfacial adhesion, and possibly better wetting of the AESO resin.

### 4.2. Hierarchical Natural Fibres Modified by Cellulose Nanocrystals (CNC) or Cellulose Nanofibrils (CNF)

**Figure 21:**
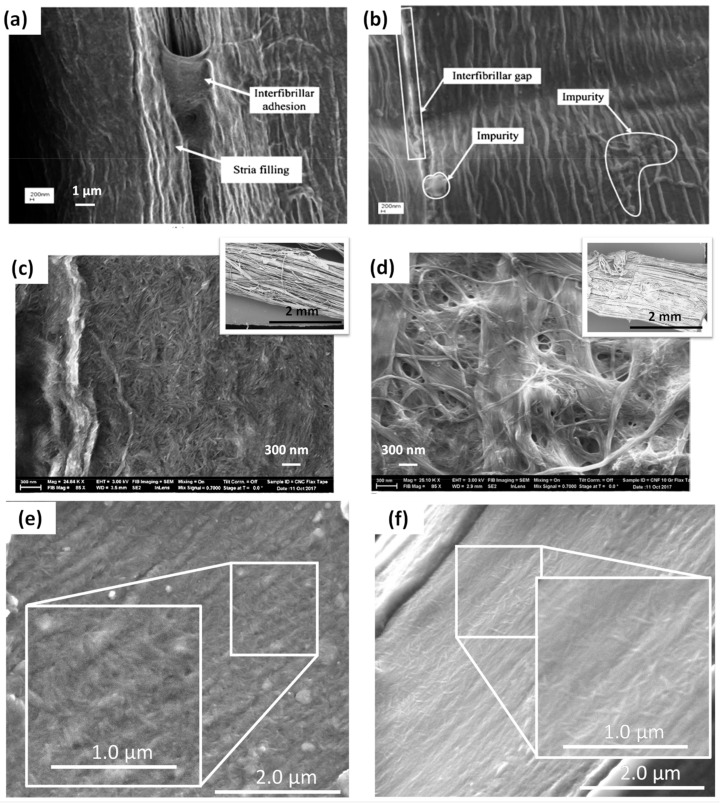
SEM Images of (**a**,**b**) nanocellulose treated hemp fibres, (**c**) CNC and (**d**) CNF 100G treated flax tapes, (**e**) CNC and (**f**) xyloglucan (XG)/CNC modified flax woven fabric (reprinted with permission from Dai and Fan (2013) [29], Ghasemi et al. (2018) [90] and Doineau et al. (2020) [91]).

Dai and Fan studied the modification of hemp yarns with the deposition of cellulose nanocrystals (CNC) at the surface of fibres [29] (Figure 21a,b).

They performed a two-step modification: (1) pre-treatment of yarns in an ultrasonic bath (60 °C, 1 h) containing a solution of cationic surfactant dodecyltrimethylammonium bromide (DTAB) at different concentrations (0.05, 0.10 or 0.15% the weight of dried fibres) and pH (10, 11, or 12); (2) dipping of DTAB pre-treated hemp fibres in a suspension of nanocellulose at 2 wt% (25 °C, 10 min). The pre-treatment at pH = 11 and DTAB 0.10% prior to the coating of hemp fibres with CNC gave the best results with an increase of the elementary fibre Young’s modulus and tensile strength of 36.1% and 72.8%, respectively. Moreover, the addition of 0.10% of DTAB resulted in greater adsorption of anionic CNC at the surface of hemp fibres. SEM images highlighted the “repairing” capacity of CNC towards hemp fibre bundles with the filling of stria at their surface and interfibrillar adhesion (Figure 21a,b).

Ghasemi et al. (2018) studied the produced modified natural fibre yarns and tapes with CNC and different types of CNF targeting textile and composite applications [90] (Figure 21c,d). As Dai and Fan [29], the authors observed the filling of gaps and the binding effect of CNC and CNF on the elementary fibres constituting the yarns/tapes. Moreover, it seems that the substrate structure, that is, yarns or tapes, induces on the distribution of CNC and CNF a more heterogeneous deposition and a larger amount of trapped nanocellulose for tapes compared to yarns. Figure 21c,d show the surfaces of two different manufactured tapes modified by the deposition of CNC and CNF 100 G (“100 G” stands for “ground for 100 min with an average particle diameter of 2.84 µm”), respectively. CNF and CNC cover the entire tape surface forming a three-dimensional network with many bridges between the fibres. Lower magnification SEM images inserted in Figure 21c,d taken at the scale of tapes and yarns show more uniform adsorption of CNC through the structure of yarns and tapes due to its smaller size compared to CNF 100G. Finally, the release of water appeared to be easier and more efficient with the presence of CNC in the structure of flax yarns/tapes. Indeed, the drying rate was almost two times faster compared to untreated yarns/tapes. However, the authors did not use these treated natural fibre yarns and tapes to manufacture flax-reinforcement-based biocomposite materials.

**Figure 22:**
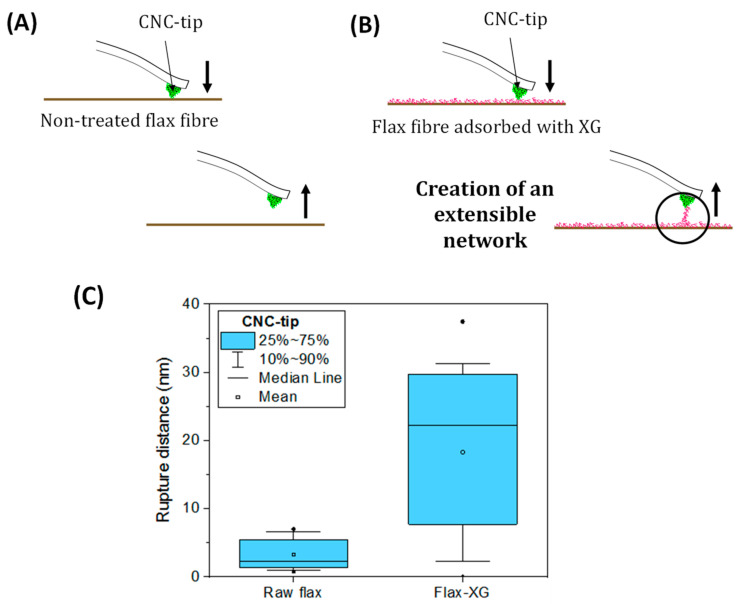
Illustration of the adhesive force measurements conducted by AFM with a CNC modified tip on two substrates: (**A**) non-treated flax fibres and (**B**) a XG modified flax fibre; and (**C**) measured rupture distances between the CNC-tip and raw flax and XG-flax substrates (reprinted with permission from Doineau et al., 2020 [91]).

Hierarchical flax fabrics were also developed by adsorbing CNC (Figure 21e) and xyloglucan (XG) on fibres (Figure 21f). XG is a flexible polysaccharide belonging to the hemicellulose family and was used as a binding agent due to its strong affinity towards cellulose surfaces [92,93]. Adsorption isotherms of CNC and XG on the surface of flax fibres were determined by UV spectroscopy with fluorescence labelled XG and CNC. The adsorbed amounts of XG and CNC on flax fibres reached a plateau at around 20 mg/g_fibres_, that is, 2 wt%. CNCs were randomly oriented on the surface of flax fibres and covered almost all surfaces with some missing areas, probably due to the complex architecture of the flax fabric made of twisted and woven yarns. Moreover, AFM force measurements were performed with a CNC modified tip on two different substrates: raw flax fibres and flax fibres treated with XG (Figure 22). The pre-adsorption of XG on the flax fibre surface increased the rupture distance with the CNC-tip by around 1000 times considering the median values, compared to the raw fibres. These results suggested that the combination of XG and CNC adsorbed on flax fibres, interacting by hydrogen bonds and van der Waals forces, allowed the creation of an extensible network at the fibre surface.

**Figure 23:**
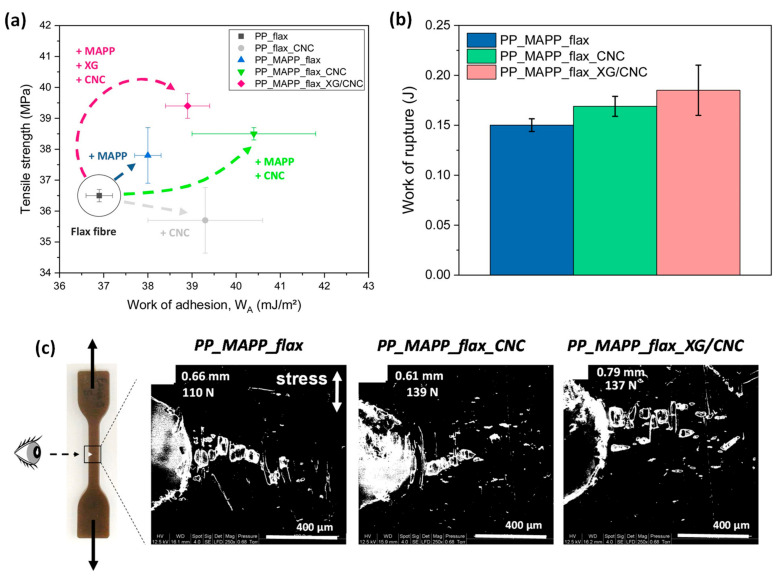
(**a**) Tensile strength by uniaxial tensile tests for the different biocomposites at 20 wt% flax fibres versus the calculated work of adhesion W_a_ (Wu approach) between flax fibres and matrix; (**b**) work of rupture of micro-tensile tests for two notched specimens with 20 wt% non-treated or CNC and XG/CNC modified flax fibres and PP/MAPP matrix with (**c**) resulted SEM observations during crack propagation in these notched specimens (reprinted with permission from Doineau et al., 2021 [94]).

Following this strategy, hierarchical short flax fibres treated by CNC and XG/CNC were used to modify the interphase zone in flax/polypropylene (PP) composites, and hence improve the load transfer from the matrix to the fibres [94]. Moreover, in some PP/flax composites, a coupling agent, that is, maleic anhydride-grafted polypropylene (MAPP), was incorporated in the matrix to promote chemical bonding with flax fibres. First, tensiometry tests with the Wilhelmy method revealed that the presence of CNC on flax fibres decreased by 29% the polar component of their surface free energy, compared to raw flax fibres. The work of adhesion and so the wettability between flax fibres and the apolar PP matrix (with or without incorporation of MAPP) was determined at 190 °C (related to processing conditions). Based on the Wu approach, it was shown that the work of adhesion increased by around 2.4% with XG/CNC, 5.4% with MAPP and XG/CNC, 6.5% with CNC and 9.5% with MAPP and CNC compared to the PP/flax composite (Figure 23a). Moreover, combining XG/CNC modified interphases with a MAPP coupling agent enhanced slightly the ultimate mechanical properties of biocomposite materials with higher uniaxial tensile strength from 36.5 MPa to 39.4 MPa compared to the PP/flax composite (Figure 23a). An increase of 23.2% of the work of rupture was also measured by micro-mechanical tensile tests, compared to PP/MAPP/flax composite (Figure 23b). Furthermore, the combination of the MAPP coupling agent and the hierarchical flax fibres modified with XG/CNC showed modifications in the failure pattern of the biocomposite as revealed by in situ micro-mechanical tensile SEM experiments and illustrated in Figure 23c. Indeed, the crack propagation was more uneven with the formation of numerous micro-cracks, compared to the quasi-linear propagation of a macro-crack for the PP/MAPP/flax composite.

The characteristics and properties of the different hierarchical biocomposites reinforced with bio-based nanoparticle modified natural fibres are summarized in Table 3. It has to be mentioned that some research works not included in Table 3 are focused on the deposition or growth of biobased nano-objects on natural fibres, but their application to composite materials by conducting wettability or (micro-) mechanical tests with polymer matrices was not considered [29,90,91]. Based on Table 3, it appears that promising results were obtained at the microscopic scale with an increase of the IFSS and/or the work of rupture. On the other hand, mechanical tests conducted at the macroscopic scale revealed more contrasting results in terms of tensile and flexural strength. This suggests that the preparation of such hierarchical biocomposites requires an optimization of the different processing steps to guarantee the efficiency of the nanostructurated interphase from the micro-scale, that is, local interfacial adhesion, up to the composite material, that is, the resulting structural performance. This is primarily due to other microstructural parameters that could be affected when incorporating hierarchical fibres in polymer matrices, that is, fibre volume fraction and porosity or alignment of fibre yarns in the case of composite laminates. Finally, the treatment procedure appears as a key, especially when considering its scale-up for composite manufacturing. The BC growth technique gives interesting results but its implementation in composite materials could be difficult due to the specific conditions required to control the fermentation bio-process, that is, the culture medium, pH, temperature, and especially the incubation time that can last for several days. In this regard, the spontaneous adsorption of CNC or CNF on fibre surfaces appears as an interesting approach more suitable for an industrial scale-up in composite or textile applications.

## 5. Conclusions

This review highlighted the key role of nano-objects in hierarchical architectures observed in biological systems such as bones, nacre, and wood. These nanoscaled-hierarchical structures increase significantly the tensile strength and toughness of biological systems by modifying the load transfer within their microstructure and the failure mechanisms in interfacial regions. Based on these findings, the bio-inspired concept of hierarchical fibres modified by nano-objects has been considered to enhance the fibre/matrix interphase in man-made composite materials. The coating of synthetic and natural fibres with nano-objects (CNTs, graphene, ZnO nanowires, nano-TiO_2_, nanoclay, BC, CNC, CNF) changes their surface properties with higher roughness and specific surface area. Moreover, nano-objects localized at the interface allow a smoother transition between the fibres and the matrix in terms of microstructure and mechanical properties of the interphase, hence improving the load transfer within the composite. Hierarchically nanostructured fibres also ensure a better mechanical interlocking with the matrix and prevent fibre debonding. Finally, nano-objects can hinder crack propagation, which follows a zig-zag path preferentially at interfaces. As a result, the incorporation of hierarchical fibres in composite materials greatly modifies their mechanical behaviour. The interfacial shear strength (IFSS) between fibres and polymer matrix is enhanced, contributing to the better mechanical performance of the composites, especially their strength and toughness. Based on this concept, fully bio-based hierarchical composites have been developed. In particular, the deposition and/or growth of various nanocelluloses on natural fibres showed promising results with more cohesive interphases resulting in higher IFSS, tensile and flexural strength as well as modified thermomechanical behaviour. A “repairing” or binding effect between elementary fibres has also been pointed out for natural fibre bundles, yarns or tapes. Nevertheless, increased voids and worst impregnation have been observed in some cases due to thick CNC layers, resulting in a loss of the mechanical properties of the biocomposites. Concluding, the bio-inspired concept of hierarchical structures appears as an interesting strategy for the development of sustainable fibre reinforced composite materials with enhanced structural properties and need to be further investigated. Developing new processes to build-up such hierarchically nanostructured fibre reinforced composite with long-scale interphases as those observed in biological systems could be the key. Besides, research works could take advantage of the specific properties of nano-objects and their functionalization to build-up functional interphases in composites with, for example, dielectric properties or sensing/response properties to temperature, relative humidity or strain/stress.

## Figures and Tables

**Table 1:** Fully synthetic hierarchical composites: characteristics and properties.

Matrix/Fibre	Nano-Objects, Treatment Method	Properties				Ref.
		Surface Properties	Wettability	IFSS (MPa)	Composite Microstructure and Properties	
**PMMA/IM7 carbon fibres**	CNTs, CVD method (growth) ▼^1^ Drawback: drop by 15–17% of CNTs-carbon fibre strength	Surface area: ▲^1^ 141%	27.4° → 21.6° Drop-on-fibre technique	▲ 12.5 MPa ± 0.2 → 15.8 MPa ± 0.4 SFFT technique	*–*	Qian et al., 2010 [57]
**Epoxy resin/GTMAC modified carbon fibres**	APTES functionalized nanoclays, cations exchange method	Roughness: 64 nm → 103 nm Coefficient of friction: ▲ 11% Surface area: ▲ 5%	~42° → ~25° Drop-on-fibre technique	▲ 24.8 MPa ± 3.5 → 32.8 MPa ± 4.5 SFFT technique	Fibres embedded tightly by matrix, crack deviation with nanoclays (based on failure surface observations)	Zabihi et al., 2017 [50]
**Epoxy resin/carbon fibres**	Graphene nanosheets (EG), liquid phase deposition	Irregular and micro-scale EG attached along the fibre axial direction	–	–	Transition phase from matrix to fibres with local stiffening (AFM, force modulation mode) ILSS: ▲ 28% Flexural modulus and strength: ▲ 5% and ▲ 31% (with 1.0 wt% EG in evaporate solvent)	Chen et al., 2015 [21]
**Epoxy resin/carboxylic acid modified aramid fibres**	ZnO nanowires, deposition method (dip-coating in a seed suspension and growth on fibre)	Presence of large crystalline ZnO nanowires on fibres, relatively uniform in length and diameter	–	▲ 11.0 MPa ± 2.5 → 16.8 MPa ± 2.8 SFFT technique	–	Ehlert and Sodano 2009 [56]

^1^ ▲ increased values or advantages; and ▼ decreased values or drawbacks.

**Table 2:** Hybrid hierarchical composites: characteristics and properties.

Matrix/Fibre	Nano-Objects, Treatment Method	Properties				Ref.
		Surface Properties	Wettability	IFSS (MPa)	Composite Microstructure and Properties	
**Natural Fibres + Synthetic/Mineral Nano-Objects**						
**PLA/Kenaf fibres**	**Exfoliated graphite nanoplatelets (xGnP)**, adsorption into acetone → excess of xGnP, not adsorbed on fibre surface, dispersed within PLA during compounding	physically-adsorbed multi-layers of xGnP on fibre surfaces	–	–	Flexural modulus: ▲^1^ 23% (with 40 wt% kenaf and 5 wt% xGnP added in acetone) ▼^1^ Flexural strength with the addition of xGnP (decrease of the interfacial adhesion)	Han et al., 2012 [64]
**PLA /sisal fibres**	**ZnO nanowires**, hydrothermal deposition method (dip-coating in a seed suspension and growth on fibre)	6 dip-coating cycles: nanowires relatively uniform with typical diameter of about 100 nm and length of ~2 µm	–	▲ 2.8 MPa → 7.2 MPa Single fibre pull-out technique ▼Drawback: very low IFSS values compared to literature	–	Yang et al., 2020 [66]
**Epoxy resin/flax yarns**	**Carboxyl-functionalised CNTs**, spray-drying process (better results for 1 wt% CNTs suspension concentration)	Uniformly dispersed and randomly oriented CNTs	–	▲ 43.7 MPa → 55.0 MPa Single yarn pull-out technique ▼Drawback: no information on test settings	ILSS: ▲ 20%	Li et al., 2015 [65]
**Epoxy resin/flax yarns**	**ZnO nanorods**, hydrothermal deposition method (dip-coating in a seed suspension and growth on fibre)	Highly oriented hexagonal ZnO nanorods	–	▼ 19.3 MPa → 12.7 MPa SFFT technique (single yarn)	Fracture surfaces: improved mechanical interlocking 3D reconstruction of fibre yarns: matrix cracks and debonding between yarns and epoxy matrix	Sbardella et al., 2021 [69]
**Epoxy resin/flax fibres**	**Nano-TiO_2_ particles**, dipping in nano-TiO_2_/KH560 suspension (optimum results with 2.34 wt% nano-TiO_2_ and higher tensile strength of single fibre)	Non-uniform distribution of nano-TiO_2_ with the presence of aggregates (150–300 nm)	–	▲ 20.7 ± 1.7 MPa → 29.3 ± 1.3 MPa Microbonding technique (single fibre)	Flexural strength and modulus: ▲ 30% and ▲ 17% resp.	Wang et al., 2015 [67]
**Synthetic Fibres + Biobased Nano-Objects**						
**Epoxy resin/glass fibres**	**BC**, dipping of fibres in BC culture medium, fermentation process	BC-based pellicles around glass fibres	~30° → 63.5° Sessile drop technique (water/air) on (BC-)glass slides ▼Drawback: no values with epoxy matrix	▲ 14.1 ± 1.9 MPa → 21.5 ± 2.2 MPa Microbonding technique (single fibre)	–	Chen et al., 2014 [79]
**Epoxy resin/chopped glass fibre roving**	**CNC**, dipping in aqueous suspension (optimum results for 1 wt% CNC)	Partial penetration of CNC within the roving	–	▲ 32.2 ± 3.8 MPa → 53.5 ± 4.3 MPa SFFT technique	Tensile properties: no improvement, Flexural strength and modulus: ▲ 39.3% and ▲42.9% resp.	Asadi et al., 2016 [80]

^1^ ▲ increased values or advantages; and ▼ decreased values or drawbacks.

**Table 3:** Hierarchical biocomposites reinforced with bio-based nanoparticle modified natural fibres: characteristics and properties.

Matrix/Fibre	Nano-Objects, Treatment Method	Properties				Ref.
		Surface Properties	Wettability	Micro-Mechanical Test, IFSS	Composite Microstructure and Properties	
**PLA/sisal fibres**	**BC**, growth on fibre surface (3–7 days, 30 °C), fermentation process ▼^1^ Drawback: long incubation time, decrease of fibre tensile strength and Young’s modulus	BC nanofibers randomly oriented on fibre surface	–	▲^1^ IFSS: 12.1 MPa ± 0.5→ 14.6 MPa ± 1.2 Single fibre pull-out technique	Longitudinal tensile strength and modulus: ▲ 44% and ▲ 42%	Juntaro et al., 2007 [84] and Pommet et al., 2008 [28]
**PLA/hemp fibres**				–	Longitudinal tensile strength and modulus: ▼ 5.2% and ▼ 33%	
**CAB/sisal fibres**				▲ IFSS:1.02 MPa ± 0.06→ 1.49 MPa ± 0.03 Single fibre pull-out technique	Longitudinal tensile strength and modulus: ▲ 8.1% and ▲ 59%	
**CAB/hemp fibres**				▲ IFSS: 0.76 MPa ± 0.06→ 1.83 MPa ± 0.12 Single fibre pull-out technique	Longitudinal tensile strength and modulus: ▼ 12% and ▼ 32%	
**PLA/sisal fibres**	**BC**, dipping in freeze-dried BC suspension (3 days) ▼ Drawback: long incubation time, decrease of fibre tensile strength and Young’s modulus	Surface area: ▲ 390% and ▲ 670% for “hairy” and “dense” BC-sisal fibres, respectively	–	–	“Dense” BC-sisal fibre: Tensile strength and modulus: ▼ 2.4% and ▲5.5% resp. Flexural strength and modulus: ▼ 6.1% and ▲ 7.0% resp. “Hairy” BC-sisal fibre: Tensile strength and modulus: ▼ 1.5% and ▲ 0.8% resp. Flexural strength and modulus: ▼ 3.4% and ▲ 2.3% resp.	Lee et al., 2012 [86]
**PP/MAPP/flax fibres**	**CNC and xyloglucan (XG)**, dipping in aqueous suspension	Homogeneous adsorption, creation of an extensible network XG/CNC at the surface of the fibre	▼ fibre polarity by 29%with CNC at the surface, ▲ work of adhesion fibre/PP by combining MAPP with XG/CNC (+ 5.4%) or CNC (+ 9.5%)	▲ 23.2% of the work of rupture with the treatment XG/CNC compared to PP/MAPP/neat flax fibres, Crack propagation more uneven with numerous micro-cracks (micro-tensile test in situ SEM)	▲ Tensile strength: 36.5 MPa → 39.4 MPa (MAPP coupling agent and XG/CNC)	Doineau et al., 2020 [91] and Doineau et al., 2021 [94]

^1^ ▲ increased values or advantages; and ▼ decreased values or drawbacks.

## Data Availability

Not applicable.
